# Physicochemical, Rheological, In-Vitro Digestibility, and Emulsifying Properties of Starch Extracted from Pineapple Stem Agricultural Waste

**DOI:** 10.3390/foods12102028

**Published:** 2023-05-17

**Authors:** Jiratthitikan Sriprablom, Manop Suphantharika, Siwaporn Meejoo Smith, Taweechai Amornsakchai, Jukkrapong Pinyo, Rungtiwa Wongsagonsup

**Affiliations:** 1 Division of Food Technology, Kanchanaburi Campus, Mahidol University, Kanchanaburi 71150, Thailand; jiratthitikan.new@gmail.com; 2 Department of Biotechnology, Faculty of Science, Mahidol University, Rama 6 Road, Bangkok 10400, Thailand; manop.sup@mahidol.ac.th; 3 Center of Sustainable Energy and Green Materials and Department of Chemistry, Faculty of Science, Mahidol University, Nakhon Pathom 73170, Thailand; siwaporn.smi@mahidol.ac.th (S.M.S.); taweechai.amo@mahidol.ac.th (T.A.); 4 Faculty of Agriculture at Kamphaeng Saen, Kasetsart University, Nakhon Pathom 73140, Thailand; jukkrapong.p@ku.th

**Keywords:** pineapple stem starch, physicochemical properties, agricultural waste, rheology, in vitro starch digestion, O/W emulsion

## Abstract

In this study, the physicochemical, rheological, in vitro starch digestibility, and emulsifying properties of starch extracted from pineapple stem agricultural waste were investigated in comparison with commercial cassava, corn, and rice starches. Pineapple stem starch had the highest amylose content (30.82%), which contributed to the highest pasting temperature (90.22 °C) and the lowest paste viscosity. It had the highest gelatinization temperatures, gelatinization enthalpy, and retrogradation. Pineapple stem starch gel had the lowest freeze–thaw stability, as evidenced by the highest syneresis value of 53.39% after five freeze–thaw cycles. Steady flow tests showed that pineapple stem starch gel (6%, *w*/*w*) exhibited the lowest consistency coefficient (*K*) and the highest flow behavior index (*n*), while dynamic viscoelastic measurements gave the gel strength in the following order: rice > corn > pineapple stem > cassava starch gel. Interestingly, pineapple stem starch provided the highest slowly digestible starch (SDS) (48.84%) and resistant starch (RS) (15.77%) contents compared to other starches. The oil-in-water (O/W) emulsion stabilized with gelatinized pineapple stem starch exhibited higher emulsion stability than that stabilized with gelatinized cassava starch. Pineapple stem starch could therefore be used as a promising source of nutritional SDS and RS, and as an emulsion stabilizer for food applications.

## 1. Introduction

Nowadays, there is a growing global interest in exploring new starch resources and developing bio-based or environmentally friendly chemical products. Consequently, a green alternative material such as starch derived from agricultural waste would be valuable for both food and non-food products due to its low cost and availability from renewable resources [1,2,3]. Starch is the most important carbohydrate source for human nutrition and a versatile natural biopolymer found in cereals and tubers. Starch also plays an important role in controlling the structure, texture, and stability of foods [4]. There are two main categories of starch polymers: amylose and amylopectin, which are homopolymers of D-glucose chain units [5]. Based on the rate and extent of starch digestion, starch is classified as rapidly digestible starch (RDS), slowly digestible starch (SDS), and resistant starch (RS) [6]. For health benefits, SDS and RS are recommended as healthy dietary carbohydrates to regulate blood glucose homeostasis and reduce glycemic response [5], which could prevent many chronic diseases such as obesity and diabetes. However, starches from different parts of plants such as leaves, stems, roots, and fruits differ significantly in terms of their morphological and physicochemical properties as well as their digestibility.

Pineapple is one of Thailand’s most commercially important fruit crops, producing more than 1.67 million tons annually [7]. Accordingly, increasing pineapple production results in a predictable amount of waste being produced each year. After pineapple harvest, the stems and leaves are considered agricultural waste. Most of this waste has no clear end-use and can be disposed of improperly, which can lead to serious environmental problems [1]. Therefore, it is crucial to utilize pineapple stem agricultural waste to improve the sustainability and economics of the process and potentially support value addition in medical and industrial food products.

According to previous studies, the pineapple stem was used as agricultural waste to obtain the enzyme bromelain, which can be used in the food and pharmaceutical industries [8,9]. Recently, pineapple stem has been used as an alternative starch source for food and non-food applications [1,2,10,11,12]. In our previous studies, the fundamental characteristics of starch extracted from pineapple stems were determined, such as extraction yield, amylose content, particle size distribution, granule morphology, X-ray diffraction, swelling power, solubility, pasting properties, and gelatinization properties compared to commercially available rice, corn, and cassava starches [1]. The extraction yield of pineapple stem starch was 30% in dry weight [1]. Pineapple stem starch granules with a median particle size of 9.69 μm had a semi-angular shape with partially round segments that had two or more flat surfaces [1]. Pineapple stem starch exhibited an A- type crystalline structure with 25.12% relative crystallinity [1,2]. However, the in vitro digestibility of pineapple stem starch has not yet been studied. Moreover, the rheological and emulsifying properties of pineapple stem starch are poorly known. Therefore, the aim of this study was to characterize the extracted pineapple stem starch and investigate its rheological properties, in vitro starch digestibility, and ability to stabilize oil-in-water (O/W) emulsions in comparison to commercially available cassava, corn, and rice starches to provide valuable information for future applications.

## 2. Materials and Methods

### 2.1. Materials

**Figure 1:**
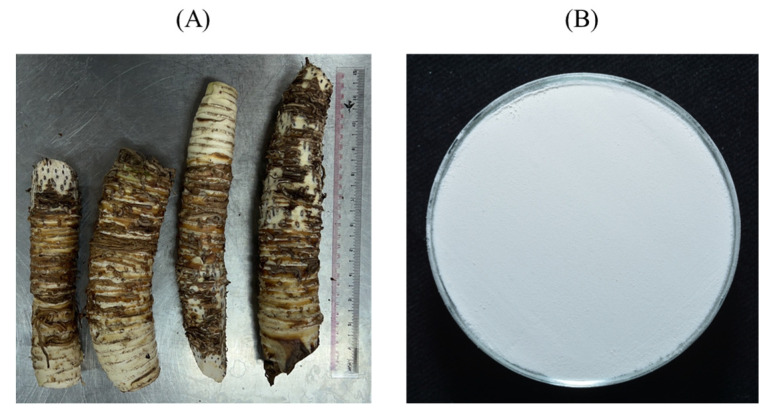
(**A**) Pineapple stems (**B**) Pineapple stem starch.

Pineapple stems (*Ananas comosus*, Smooth Cayenne variety) were collected as agricultural waste after harvest at a pineapple farm (Kanchanaburi, Thailand) (Figure 1A). All freshly collected stems from the pineapple farm (10 kg) were immediately brought to the laboratory within a day and stored in the freezer until used for starch isolation. The stale stems were not selected for this study. Cassava starch (Jade leaf brand), corn starch (Kruawangthip brand), and soybean oil were purchased from a local market (Bangkok, Thailand). Rice starch was provided by Cho Heng Rice Vermicelli Factory Co., Ltd. (Nakhon Pathom, Thailand). Porcine pancreatin (P7545, 8xUSP specifications) and amyloglucosidase (A-7095, 300 U/mL) were obtained from Sigma-Aldrich Chemical Co. (St. Louis, MO, USA). The glucose oxidase/peroxidase (GOPOD) assay kit was obtained from Megazyme International Ireland Ltd. (Wicklow, Ireland). All other chemicals used in the study were analytical grade.

### 2.2. Extraction of Pineapple Stem Starch

Extraction of pineapple stem starch was prepared according to the methods of Nakthong et al. [1] and Tangsrianugul et al. [13] with some modifications. The pineapple stems were washed, peeled, and cut into small pieces. These pieces were ground with 400 ppm sodium metabisulfite solution (Na_2_S_2_O_5_) at a weight ratio of 1:3 in a mill. The pineapple stem slurry was then stirred at room temperature for 18 h. The slurry was then filtered with a filter cloth to remove coarse fibrous material and then filtered through 100, 140, and 270- mesh screens, respectively, to remove fine fibrous material. Starch in the filtrate fraction was collected by centrifugation at 3000× *g* for 10 min. The liquid fraction was decanted, and the yellow layer on the surface of the sediment was scraped off manually. The starch sediment was washed several times with distilled water until the supernatant was clear. The starch cake was dried overnight in an oven at 45 °C. Finally, the dried pineapple stem starch was ground and sieved through a 100-mesh sieve (Figure 1B). The isolated pineapple stem starch was kept in a polyethylene zipper bag and stored at room temperature. The pH of the isolated pineapple stem starch was 6.79.

### 2.3. Physicochemical Properties of Starches

#### 2.3.1. Amylose Content

The amylose content of pineapple stem starch and commercial starches (cassava, corn, and rice starches) was analyzed using an amylose/amylopectin test kit (Megazyme International Ireland Ltd., Wicklow, Ireland). Starch samples were defatted by heating them in dimethyl sulfoxide (DMSO). Lipids were removed by precipitating the dispersed starch in 95% (*v*/*v*) ethanol. The precipitated starch was dissolved in an acetate/salt solution and subsequently used for amylose determination. The lectin concanavalin A (Con A) was added to the starch suspension to precipitate amylopectin, which was removed by centrifugation. Glucose obtained from the enzymatic hydrolysis of amylose in the supernatant was analyzed using a GOPOD reagent.

#### 2.3.2. Pasting Properties

Pineapple stem starch and commercial starches were analyzed for pasting characteristics using a Rapid Visco-Analyzer (RVA) (Model-4, Newport Scientific Pty. Ltd., Warriewood, Australia) based on a standard 1 profile. The 28 g starch suspension was prepared by mixing the starch sample with distilled water to obtain a final starch concentration of 6% *w*/*w*, and stirred in an aluminum container for 20 s before being introduced into the instrument. The Rapid Visco Unit (RVU) was used to express the viscosity.

#### 2.3.3. Visual Observation of Starch Pastes

The pineapple stem and commercial starch pastes obtained from the RVA measurement were placed on a glass plate and cooled to 25 °C. All starch pastes were photographed with a digital camera (Kodak EasyShare M340, 10.2 megapixels, Rochester, NY, USA) against a black background to qualitatively evaluate their appearance and clarity.

#### 2.3.4. Microscopic Observation of Starch Pastes

Prior to microscopic observation, the starch pastes from the RVA were stained with 0.1% iodine solution, transferred to a slide, and covered with a coverslip. The morphology of the pastes was observed with a normal light microscope (Olympus BX51, Olympus Optical Co. Ltd., Tokyo, Japan) at 40× magnification.

#### 2.3.5. Determination of Freeze—Thaw Stability

Five grams of starch paste from the RVA were placed in a 15-mL centrifuge tube, frozen at −18 °C for 21 h, and then thawed in a water bath at 30 °C for 3 h, which was repeated for five cycles. Percent syneresis was determined for each freeze–thaw cycle by dividing the weight of water separated after centrifugation of the sample tube at 2200× *g* for 10 min by the initial gel weight and multiplying it by 100.

#### 2.3.6. Determination of Thermal Properties 

The starch samples were analyzed for their thermal properties using a differential scanning calorimeter (DSC) (DSC-1 STARe System, Mettler-Toledo AG, Schwerzenbach, Switzerland) as described by Wongsagonsup et al. [14]. An aluminum pan containing a mixture of 3 mg (dry weight) starch sample and 9 mg distilled water was hermetically sealed and equilibrated at room temperature for 1 h before being placed in the DSC instrument. The sample pan was heated from 25 to 100 °C at a heating rate of 10 °C/min. For retrogradation analysis, the gelatinized starch from DSC measurement was stored at 4 °C for 7 days and then analyzed by DSC under the same conditions as for starch gelatinization. The percentage of retrogradation was calculated as follows:

Retrogradation (%) = (∆*H*_ret_/∆*H*_gel_) × 100

where ∆*H*_ret_ is the retrogradation enthalpy and ∆*H*_gel_ is the gelatinization enthalpy.

### 2.4. Rheology Measurement

The freshly prepared starch gels from the RVA were analysed for their rheological properties at 25 °C using a rheometer (HAAKE MARS 40, Thermo Fischer Scientific, Karlsruhe, Germany) equipped with a cone and plate sensor (2° cone angle, 40 mm diameter, and 0.1 mm gap). For the dynamic viscoelastic measurements, a linear viscoelastic range was determined by varying a strain of 0.01–100% at a fixed frequency of 10 rad/s. A dynamic frequency sweep test was then performed in the linear viscoelastic range with a constant strain of 0.2% in a frequency range of 0.1–10 rad/s. The storage modulus (*G*′), loss modulus (*G*″), and loss tangent, defined as the ratio of *G*″ to *G*′ (tan *δ*), were then plotted as a function of frequency.

For steady flow measurements, samples were sheared continuously from 0.1 s^−1^ to 300 s^−1^ in 1 min followed by an immediate reduction from 300 s^−1^ to 0.1 s^−1^ in the next 1 min. The obtained flow curves were analyzed using the Herschel-Bulkley model as follows:

*σ* = *σ*_0_ + *Kγ̇ ^n^*

where, *σ* is the shear stress (Pa), *σ*_0_ is the yield stress (Pa), *γ̇* is the shear rate (s^−1^), *K* is the consistency coefficient (Pa.s*^n^*), and *n* is the flow behavior index (dimensionless).

### 2.5. In-Vitro Starch Digestibility

Starch digestibility was determined in vitro according to the method of Englyst et al. [6] with slight modifications. Before testing, the starch samples were boiled at 100 °C for 15 min. To determine in vitro starch digestibility, aliquots of digested starch were taken after enzymatic digestion with an enzyme mixture of porcine pancreatin and amyloglucosidase at 20 and 120 min. Glucose content released after 20 min (G20) and 120 min (G120) of enzymatic digestion was measured using a GOPOD assay kit. The starch fractions were calculated as follows:

Rapidly digestible starch (RDS) (%) = %G20 × 0.9

Slowly digestible starch (SDS) (%) = (%G120 − %G20) × 0.9

Resistant starch (RS) (%) = 100 − (%RDS + %SDS)

### 2.6. Determination of Emulsifying Properties 

The emulsion was prepared according to the method of Sriprablom et al. [15]. The starch suspension containing 5% (*w*/*w*) starch in distilled water was boiled in a water bath at 100 °C for 15 min. Emulsions were prepared by mixing 10% (*w*/*w*) soybean oil and 90% (*w*/*w*) aqueous phase of the cooked starch suspension after cooling to room temperature in a disperser (model Ultra Turrax T18, IKA Works, Inc., Wilmington, NC, USA) at 10,000 rpm for the first 1 min and then at 14,000 rpm for an additional 4 min. Sodium azide was used as a preservative at a concentration of 0.02% (*w*/*w*). The freshly prepared emulsions (10 mL) were filled into glass vials and stored at room temperature for 15 days. The stability of the emulsion was evaluated by the creaming index (CI), defined as follows:

CI (%) = (*H*_S_/*H*_E_) × 100

where *H*_S_ is the height of the serum layer and *H*_E_ is the total height of the emulsions.

### 2.7. Statistical Analysis

All measurements were performed in three replicates. Results were expressed as means ± standard deviations. Statistical analyses were carried out using the SPSS version 26.0 program for Windows (SPSS Inc., Chicago, IL, USA). A significant difference between means was analyzed using one-way ANOVA and Turkey’s multiple range test at 5% significance level (*p* ≤ 0.05).

## 3. Results and Discussion

### 3.1. Amylose Content

Amylose is one of the key components that significantly influences the physicochemical properties and starch digestibility [1,16]. The amylose contents of pineapple stem, cassava, corn, and rice starches are presented in Table 1. Pineapple stem starch had the highest amylose content, which is similar to the value of 34.37% reported by Nakthong et al. [1], but higher than the value reported by Rinju and Harikumaran-Thampi [2] (23.86%). Consequently, the variation of amylose content with respect to the same starch type depends mainly on the environment, genotype, and botanical sources. The amylose contents of cassava, corn and rice starches agreed with the data reported by Srichuwong and Jane [17]. Normally, root and tuber starches have lower amylose content than normal cereal starches. Starches with high amylose content help to delay starch digestion and lower blood glucose levels [16,18], which is useful for the production of low glycemic index foods. 

### 3.2. Pasting Properties

**Figure 2:**
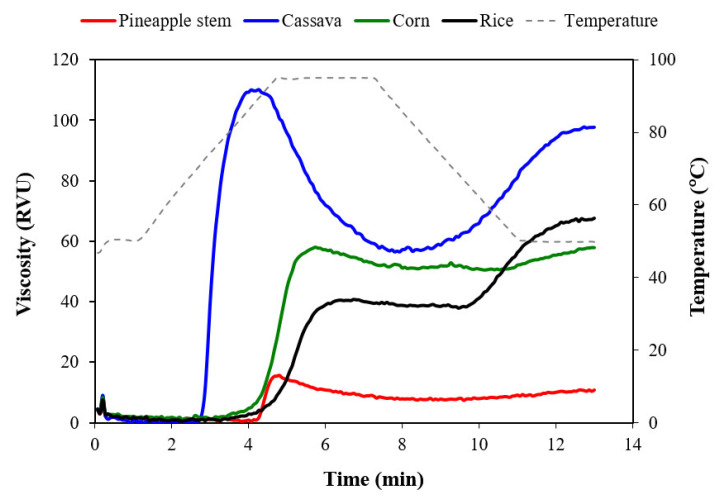
RVA pasting curves of pineapple stem, cassava, corn, and rice starches with 6% (*w*/*w*) starch slurry.

Figure 2 and Table 2 show the RVA pasting curves and pasting parameters of pineapple stem starch compared with commercially available cassava, corn, and rice starches, respectively. In accordance with the results, pineapple stem starch exhibited the lowest peak viscosity, lowest trough, lowest final viscosity, and lowest setback, but the highest pasting temperature. The breakdown of pineapple stem starch was higher than that of rice starch but lower than that of cassava starch. The higher amylose content of starch correlated with lower peak and breakdown viscosities and higher pasting temperatures [17]. However, the lipids present in rice starch can form a helical complex with the amylose, which enhances the interactions of the starch molecules, resulting in the lowest breakdown value of rice starch. A similar trend was observed by Nakthong et al. [1], who studied the pasting behavior of pineapple stem starch. The pineapple stem starch exhibited very low paste viscosity, indicating that the starch granules were very strong and their swelling was very limited. The lower peak, trough, and final viscosities of the starch could be due to more restricted starch granules [19]. Limited swelling of pineapple stem starch granules limited leaching of amylose molecules from the granules, resulting in less amylose aggregation during cooling and consequently lower setback viscosity. The limited swelling of the granules and viscosity can be attributed to the highest amylose content of the pineapple stem starch (Table 1). Amylose enhances the interaction between starch molecules, which maintains the integrity of starch granules and increases their stability [20,21]. Consequently, higher amylose content could suppress the swelling ability of the granules and reduce the pasting behavior of starch. Conversely, cassava starch exhibited the highest peak viscosity and breakdown but the lowest pasting temperature (Table 2), corresponding to the lowest amylose content (Table 1). The low amylose content may contribute to the high viscosities and low pasting temperature of the starch pastes [22,23].

### 3.3. Visual Appearance and Microscopic Observation of Starch Pastes

**Figure 3:**
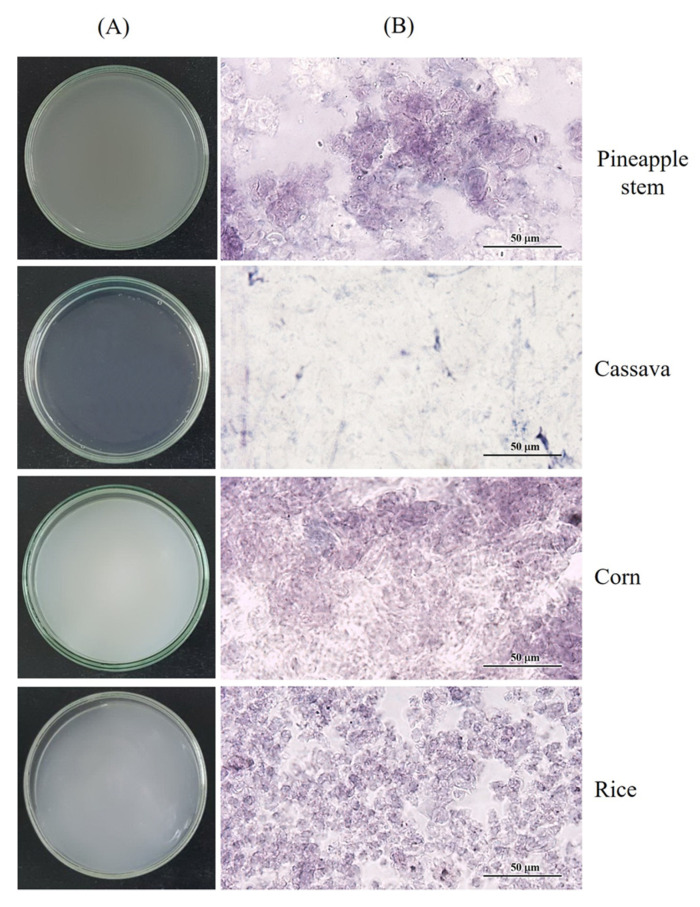
Visual appearance (**A**) and morphological characteristics (**B**) of pineapple stem, cassava, corn, and rice starch pastes from RVA measurement. The morphology of the iodine-stained starch pastes was observed with a light microscope at 400× magnification.

The visual appearance and morphological characteristics with iodine staining of pineapple stem starch paste from RVA measurement compared to cassava, corn, and rice starch pastes are shown in Figure 3. Photographs of the starch pastes showed that the cassava starch paste was transparent and clear (Figure 3A), indicating that the starch granules were completely disintegrated after gelatinization and no swollen granules remained (Figure 3B). This result agrees with that of Błaszczak and Lewandowicz [24], who found that complete dispersion of gelatinized cassava starch without swollen granules was observed at 90 °C, indicating a high swelling characteristic. In contrast, the pineapple stem, corn, and rice starch pastes with higher amylose content were turbid and opaque (Figure 3A). These starch granules did not completely disrupt but retained their granule structure, as shown in Figure 3B. This is because amylose molecules interact with amylopectin molecules to improve the stability and integrity of the granules [1,21], which can limit starch granule swelling and result in an opaque paste. In general, normal cereal starches develop turbid and opaque pastes due to the helical complexes formed between amylose molecules and lipids, which enhance the interaction between entangled amylose and amylopectin molecules, again limiting the swelling of normal cereal starches [17]. For pineapple stem, corn and rice starch pastes, the dark blue color of the amylose-iodine complex was observed in the vicinity of the swollen granules with the red-purple color of the amylopectin-iodine stain, indicating the leakage of amylose from the granules during gelatinization [24]. 

### 3.4. Freeze–Thaw Stability

The term “freeze–thaw stability” refers to the ability of starch to withstand the unfavorable physical changes that occur during freezing and thawing. Syneresis is the process by which unbound water is readily released from the polymeric network during thawing [25]. It is assumed that the syneresis of starch gel is directly related to the retrogradation of starch [25,26]. The results showed that all starch gels exhibited a significant increase in syneresis with increasing freeze–thaw cycles (*p* ≤ 0.05) (Table 1). Repeated freeze–thaw cycles lead to greater syneresis of the starch gel [17]. Compared to commercial cassava, corn, and rice starches, freeze–thawed pineapple stem starch gel exhibited the highest syneresis at each freeze–thaw cycle. The syneresis of pineapple stem starch gels was 19.52% after the first cycle and significantly (*p* ≤ 0.05) increased to 51.52% after three freeze–thaw cycles, and then changed little (53.21–53.39%) in cycles 4–5. While the syneresis of cassava starch gel was not observed after the first cycle and was 23.16% after five freeze–thaw cycles, which was the significantly lowest syneresis. Syneresis occurs in freeze–thaw treatments due to starch crystallization (retrogradation). Amylose retrogrades much faster than amylopectin due to its predominantly linear structure [17]. Therefore, syneresis of starch gels has been associated with the amylose content of starch. Cassava starch with the lowest amylose content (18.98%) exhibited the lowest syneresis but the highest freeze–thaw stability. Therefore, the highest syneresis of pineapple stem starch was associated with the highest amylose content (Table 1), resulting in poor freeze–thaw stability. The restricted swelling ability of the pineapple stem starch could also affect the syneresis of the starch gel. A large amount of free water surrounding the limited swollen granules can be easily released as syneresis during freeze–thaw cycles.

### 3.5. Gelatinization and Retrogradation Properties

**Figure 4:**
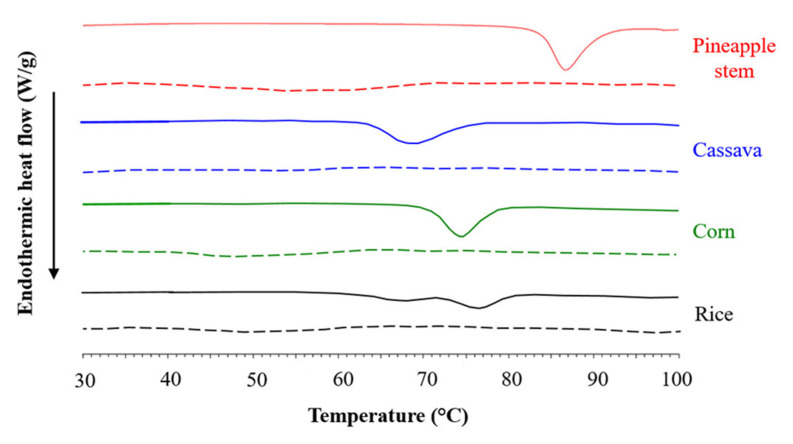
Differential scanning calorimetry (DSC) thermograms of native (solid line) and retrograded (dashed line) pineapple stem, cassava, corn, and rice starches.

The DSC thermograms of the gelatinized and retrograded starches are shown in Figure 4, and the thermal parameters are listed in Table 3. The pineapple stem starch exhibited the highest gelatinization parameters (onset, peak, and conclusion gelatinization temperatures as well as gelatinization enthalpy) compared to commercial starches (Table 3). High transition temperatures could be due to a high content of amylose, which provides structural stability and increases the granule’s resistance to gelatinization [1]. The gelatinization temperatures are highly positively correlated with the amylose content [23]. The Δ*H*_gel_ value indicates the loss of molecular order inside the granule and reflects the total crystallinity of the starch granules both in terms of the quality and quantity of the starch crystals [27]. It can be noted that pineapple stem starch is structurally more stable than cassava, corn, and rice starches, possibly due to the high amylose content [1]. Amylose molecules strongly interact with amylopectin molecules to improve the integrity of starch granules [21]. Therefore, starches with a high amylose content usually require a higher temperature to complete the cooking process.

In the case of retrograded starch, the transition temperatures (*T*_o_, *T*_p_, and *T*_c_) and melting enthalpy (Δ*H*_ret_) of the retrograded starch gels were lower than the gelatinization temperatures and Δ*H*_gel_ of the native starch counterparts. The crystallites and ordered structure of the amorphous region of retrograded starch are not as perfect as those of native starch [15]. The retrograded pineapple stem starch had the highest *T*_o_, *T*_p_, *T*_c_, and Δ*H*_ret_ values, resulting in the highest degree of retrogradation (46.07%) compared to retrograded cassava, corn, and rice starches. Retrogradation enthalpy was reported to be positively correlated with amylose content [28]. High amylose content promotes starch retrogradation. In the early stage of retrogradation, amylose rapidly and permanently reassociates to form crystal nuclei, while amylopectin slowly recrystallizes in a later stage, so that the starch molecules pass from a disordered state to a more ordered state [29,30]. Most studies have focused on how amylose content affects starch retrogradation as it affects the quality, acceptability, and shelf life of starchy foods [28,30,31]. It was also found that retrogradation results were related to those of syneresis values after freeze–thaw cycles (Table 1). At a higher retrogradation rate, the hydrogen bonds between the starch chains increased and more water was squeezed out of the gel, resulting in a higher syneresis rate [30].

### 3.6. Rheological Properties

**Figure 5:**
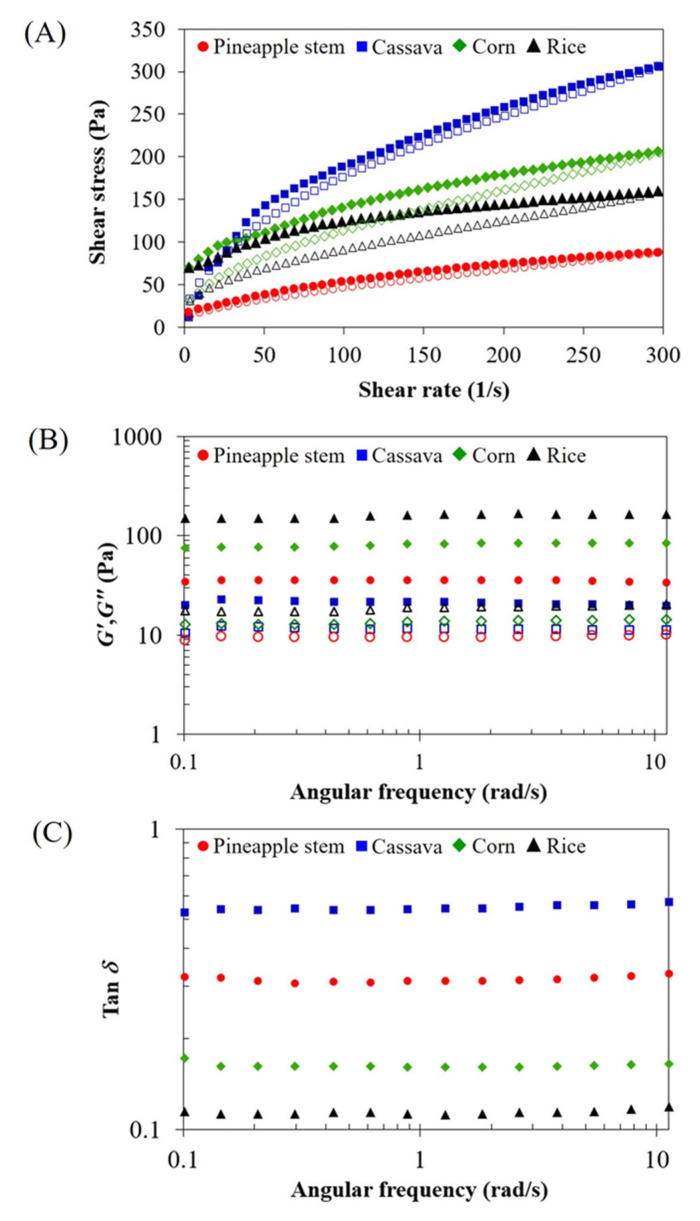
Steady flow curves (**A**), i.e., shear stress as a function of shear rate, where closed symbols represent upward curves and open symbols represent downward curves, and dynamic viscoelastic mechanical spectra, and (**B**), i.e., storage modulus, *G*′ (closed symbols) and loss modulus, *G*″ (open symbols) as a function of frequency, and dynamic mechanical loss tangent (tan *δ*) as a function of frequency (**C**) of 6% (*w*/*w*) pineapple stem, cassava, corn, and rice starch gels. All measurements were performed at 25 °C.

The rheological behavior, including steady flow characteristics, mechanical spectra (*G*′ and *G*″ as a function of frequency), and tan *δ* as a function of frequency of starch gels is shown in Figure 5. All starch gels exhibited primarily time-dependent shear-thinning (thixotropic) behavior with yield stress (*σ*_0_) for the range of shear rates used in this study (Figure 5A). The flow curves of freshly prepared starch gels can be fitted using the Herschel–Bulkley model. Table 4 provides a summary of the hysteresis loop area, the yield stress (*σ*_0_), the consistency coefficient (*K*), the flow behavior index (*n*), and the coefficient of determination (*R*^2^) of the upward flow curves. All starch gels exhibited a clockwise hysteresis loop area, indicating structural breakup caused by the shear field and a change in an existing structure, which then continues to exhibit a shear thinning characteristic during subsequent shear sweeps [32]. The larger area of the hysteresis loop, i.e., the higher thixotropy, is attributed to the greater structural breakdown of the starch gel during shearing [33]. All starch gels had *n* values of less than 1, indicating their pseudoplastic shear thinning behavior. In this study, the pineapple stem starch gel exhibited the lowest *K* value and hysteresis loop area, as well as the highest *n* value, indicating the lowest viscous properties, the highest shear resistance and structural recovery, and the lowest degree of shear thinning. The cassava starch gel had the highest *K* value and the lowest *σ*_0_ and *n* values, indicating the greatest viscous properties, the least structure formation of the gel, and the highest degree of shear thinning. Similar observations were reported by Wongsagonsup et al. [14] who studied the rheological properties of native and cross-linked cassava starch gels. They found that the limited swollen granules and high shear resistance of the highly crosslinked cassava starch gel resulted in a lower *K* value and hysteresis loop area than native cassava starch gel with complete gelatinization, and no swollen granules remained. From these results, it can be concluded that the steady flow properties, i.e., the *K* value, of all starch gels, correspond to the results of the pasting properties (Table 2).

The mechanical spectra of all starch gels showed that *G*′ and *G*″ did not cross and that *G*′ was higher than *G*″ over the entire frequency range investigated, indicating the typical gel character (Figure 5B). Pineapple stem starch gel had a lower *G*′ value than rice and corn starches, confirming the soft characteristic of pineapple stem starch gel. The limited swelling ability of pineapple stem starch could hinder the formation of a strong gel network. The corn and rice starch gels were more rigid and exhibited higher *G*′ values compared to pineapple stem starch gel. On the other hand, cassava starch gel had the lowest *G*′ value, indicating the softest gel. In the cassava starch gel with the lowest amylose content, the starch molecules are less prone to reassociate and form a strong gel. These results can be confirmed by the tan *δ* value (Figure 5C), which is the ratio of *G*″ to *G*′ and was used to explain the changes in viscoelasticity. All starch gels tested exhibited tan *δ* < 1, indicating typical gel network formation [34]. The tan *δ* value of all starch gels decreased in the following order: cassava > pineapple stem > corn > rice starch gel. It was found that the tan *δ* value of all starch gels is inversely related to the corresponding *σ*_0_ value. The lowest tan *δ* value of rice starch gel is associated with the highest *σ*_0_ value, while the highest tan *δ* value of cassava starch gel is associated with the lowest *σ*_0_ value. The tan *δ* value of pineapple stem starch gel was comparatively higher than that of corn and rice starch gels, indicating a more liquid-like behavior, which could be due to its limited swelling ability. It was concluded that the pineapple stem starch gel was more fluid and exhibited a less rigid gel than corn and rice starches. However, the gel from cassava starch exhibited the highest tan *δ* value, indicating the most liquid-like behavior of the weakest gel, which could be due to the lowest amylose content. The rheological properties of starch gel are significantly influenced by the granule size, shape, rigidity, swelling ability, amount, and type of amylose and amylopectin, as well as complexes with other components [2].

### 3.7. In Vitro Starch Digestibility

The RDS, SDS, and RS contents of the cooked starch samples are shown in Table 5. With the exception of pineapple stem starch, all cooked starch samples had the significantly highest percentage of RDS content, especially cassava starch (79.34%), which could be explained by the complete disruption of the granule structure during cooking, facilitating susceptibility to enzymatic hydrolysis [15]. Previous studies have shown that the RDS fraction is the major component in cooked native starch [15,35,36]. Interestingly, the pineapple stem starch had the highest proportion of the SDS fraction. The restricted swollen granules of pineapple starch may not be easily digested by amylolytic enzymes and may consequently delay the digestibility of starch. Compared to cassava, corn, and rice starches, pineapple stem starch had the highest SDS and RS content but the lowest RDS content, which was found for the first time in this study. This is probably related to the high amylose content of pineapple stem starch, which has the ability to inhibit starch hydrolysis by limiting starch swelling. A starch with high amylose content is more resistant to enzymatic hydrolysis by enhancing the starch’s ability to resist enzymatic digestion [37]. Apparent amylose content is negatively correlated with RDS but positively correlated with SDS and RS [20]. In addition, the fine structure of amylopectin affects the digestibility of starch. The RS content correlates positively with the average amylopectin branch chain length and the proportion of long branch chains, but negatively with the proportion of shorter branch chains [20,38]. By lowering blood cholesterol and triglyceride levels and reducing fat accumulation, RS is crucial for maintaining good health. However, higher levels of SDS may also be beneficial for human health. SDS is usually considered the most preferred dietary starch because it is completely but slowly digested in the small intestine [39]. In addition, SDS is likely associated with beneficial health effects such as improved satiety and mental performance, blood sugar control, and diabetes management [5,39,40]. 

### 3.8. Stability of Starch-Stabilized O/W Emulsions

**Figure 6:**
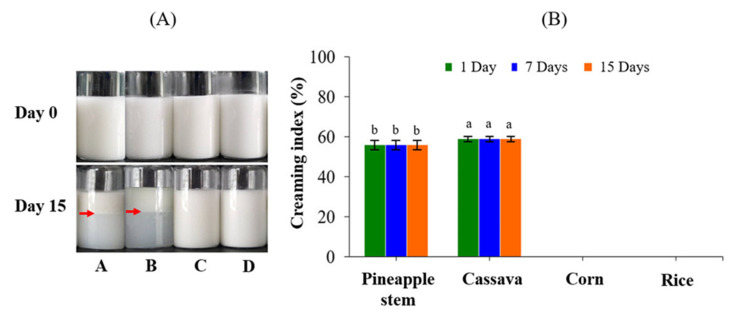
(**A**) Photographs of the 0- and 15-day stored O/W emulsions stabilized by gelatinized starch samples, with the red arrow marking the cream-serum boundary (A, pineapple stem starch; B, cassava starch; C, corn starch; D, rice starch), and (**B**) the creaming index of O/W emulsions stabilized by gelatinized pineapple stem, cassava, corn, and rice starches during storage at room temperature for 15 days. Values with different superscripts (a,b) are statistically different (*p* ≤ 0.05).

Figure 6 shows the oil-in-water (O/W) emulsions stabilized by gelatinized starch samples. The creaming stability of emulsions is evaluated by means of the creaming index (CI). As shown in Figure 6A, the emulsions stabilized with corn and rice starches aged for 15 days exhibited a homogeneous appearance with no creaming (CI = 0) (Figure 6B), indicating high emulsion stability. A lower CI is related to greater emulsion stability. A more solid-like behavior of corn and rice starch gels (Figure 5B,C) could thicken the continuous aqueous phase and stabilize the O/W emulsions. The emulsions stabilized with gelatinized corn and rice starches exhibited a viscoelastic solid or gel-like texture from the third day of storage. Some gelatinized native starches such as non-waxy rice starch, waxy rice starch, and waxy corn starch showed O/W emulsifying ability by interfacial absorption [41]. However, the emulsifying ability was not clearly related to the amylose content, the crystalline pattern, and the thermal properties of the starch [41]. On the other hand, emulsions stabilized with cassava and pineapple stem starches showed high CI values (58.91% and 55.81%, respectively) from the first day of storage to 15 days of storage. A similar result for emulsions stabilized with gelatinized native cassava starch was observed in the study of Sriprablom et al. [15]. This is probably due to the fact that a large number of hydrophilic -OH groups of completely gelatinized cassava starch could limit the emulsifying properties. In the case of the emulsion stabilized with pineapple stem starch, the CI was also high, but the lower serum layer was slightly cloudier than that of the emulsion stabilized with cassava starch (Figure 6A), indicating the presence of a small amount of suspended individual oil droplets and/or swollen starch granules. The limited swollen pineapple stem starch granules can slightly stabilize the emulsion as a particle stabilizer but are not strong enough to prevent creaming, which may be due to the hydrophilic moieties and low viscosity of the continuous aqueous phase containing swollen pineapple stem starch granules, which is similar to the result reported by Kasprzak et al. [41] for high amylose corn starch. To prevent creaming, an emulsifier must be added to the continuous aqueous phase or the native starch must be modified to increase its amphiphilic properties and produce a stable emulsion [15,41,42]. For example, starch chemically modified with octenyl succinic anhydride (OSA) has been used as an emulsifier to stabilize conventional O/W emulsions. It has been reported that OSA-modified pineapple starch has better emulsifying properties than native starch and can be used as an emulsion stabilizer in food and pharmaceutical applications [43].

## 4. Conclusions

Pineapple stems are agricultural waste that can be used as an alternative source of starch. When starch extracted from pineapple stem was compared with commercially available cassava, corn, and rice starches, pineapple stem starch exhibited special characteristics in that it had the highest amylose content, pasting, and gelatinization temperatures, gelatinization enthalpy, retrogradation and syneresis rates, and the lowest paste viscosity, indicating high granule stability. Due to its limited swelling ability, the pineapple stem starch gel had the lowest consistency coefficient and hysteresis loop area and had more liquid-like behavior than corn and rice starches. Compared with commercial starches, it had the highest contents of slowly digestible starch (SDS) and resistant starch (RS). The emulsifying property of pineapple stem starch was better than that of cassava starch. Because of these properties, pineapple stem starch could be used as a good source of nutritional SDS and RS for food applications. Pineapple stem starch with a high content of SDS and RS can be used in the formulation of a wide range of low-moisture foods such as pasta, bread, cookies, cakes, and breakfast cereals to enhance health benefits.

## Figures and Tables

**Table 1:** Amylose content and freeze–thaw stability of pineapple stem, cassava, corn, and rice starches.

Sample	Amylose Content (%)	Syneresis (%)				
		1 Cycle	2 Cycles	3 Cycles	4 Cycles	5 Cycles
Pineapple stem	30.82 ± 0.85 ^a^	19.52 ± 0.83 ^aD^	29.76 ± 0.77 ^aC^	51.52 ± 0.96 ^aB^	53.21 ± 0.74 ^aA^	53.39 ± 0.93 ^aA^
Cassava	18.98 ± 0.22 ^d^	nd *	3.82 ± 0.30 ^dD^	13.21 ± 0.84 ^dC^	21.40 ± 0.84 ^dB^	23.16 ± 0.86 ^dA^
Corn	22.88 ± 0.23 ^b^	4.15 ± 0.13 ^cD^	20.79 ± 0.46 ^bC^	31.21 ± 1.06 ^bB^	40.98 ± 0.59 ^bA^	41.13 ± 0.41 ^bA^
Rice	21.46 ± 0.32 ^c^	7.87 ± 0.27 ^bE^	16.07 ± 0.65 ^cD^	29.07 ± 0.66 ^cC^	33.28 ± 0.53 ^cB^	35.37 ± 0.74 ^cA^

* Not detected. Values in the same column with different lower-case superscript letters (a–d) are significantly different (*p* ≤ 0.05). Values in the same row with different upper-case superscript letters (A–E) are significantly different (*p* ≤ 0.05).

**Table 2:** RVA pasting parameters of pineapple stem, cassava, corn, and rice starches.

Sample	Peak Viscosity (RVU)	Trough (RVU)	Breakdown (RVU)	Final Viscosity (RVU)	Setback (RVU)	Pasting Temperature (°C)
Pineapple stem	15.78 ± 0.13 ^d^	8.47 ± 0.13 ^d^	7.31 ± 0.05 ^b^	10.53 ± 0.48 ^d^	2.06 ± 0.38 ^d^	90.22 ± 0.53 ^a^
Cassava	111.61 ± 1.47 ^a^	57.06 ± 0.49 ^a^	54.55 ± 1.10 ^a^	98.17 ± 0.60 ^a^	41.11 ± 0.50 ^a^	70.86 ± 0.29 ^c^
Corn	57.06 ± 0.96 ^b^	49.61 ± 0.79 ^b^	7.45 ± 0.13 ^b^	57.53 ± 0.42 ^c^	7.92 ± 0.44 ^c^	81.84 ± 0.82 ^b^
Rice	40.55 ± 0.48 ^c^	37.64 ± 0.48 ^c^	2.91 ± 0.00 ^c^	67.47 ± 0.73 ^b^	29.83 ± 0.30 ^b^	82.87 ± 0.83 ^b^

Values in the same column with different superscripts are significantly different (*p* ≤ 0.05).

**Table 3:** Thermal properties of pineapple stem, cassava, corn, and rice starches.

Sample	Native Starch				Retrograded Starch *				Retrogradation (%) **
	*T*_o_ (°C)	*T*_p_ (°C)	*T*_c_ (°C)	Δ*H*_gel_ (J/g)	*T*_o_ (°C)	*T*_p_ (°C)	*T*_c_ (°C)	Δ*H*_ret_ (J/g)	
Pineapple stem	83.77 ± 0.09 ^a^	86.53 ± 0.13 ^a^	90.90 ± 0.15 ^a^	17.24 ± 0.10 ^a^	42.35 ± 0.95 ^a^	59.68 ± 0.45 ^a^	70.45 ± 0.60 ^a^	7.94 ± 0.07 ^a^	46.07 ± 0.43 ^a^
Cassava	63.23 ± 0.22 ^d^	68.59 ± 0.09 ^d^	75.80 ± 0.23 ^d^	15.48 ± 0.12 ^b^	39.20 ± 0.11 ^b^	53.67 ± 0.34 ^b^	68.02 ± 0.69 ^b^	2.98 ± 0.03 ^d^	19.23 ± 0.18 ^d^
Corn	70.29 ± 0.23 ^b^	74.08 ± 0.25 ^c^	78.83 ± 0.35 ^c^	14.33 ± 0.25 ^c^	41.50 ± 0.27 ^a^	52.09 ± 0.42 ^c^	64.23 ± 0.11 ^c^	5.26 ± 0.02 ^b^	36.71 ± 0.68 ^b^
Rice	64.91 ± 0.37 ^c^	76.24 ± 0.08 ^b^	80.89 ± 0.46 ^b^	11.12 ± 0.55 ^d^	41.75 ± 0.29 ^a^	52.30 ± 0.27 ^c^	63.40 ± 0.17 ^d^	3.86 ± 0.13 ^c^	34.73 ± 0.81 ^c^

* After storage for 7 days at 4 °C; ** Retrogradation (%) = (Δ*H*_ret_**/**Δ*H*_gel_) × 100. Values in the same column with different superscripts are significantly different (*p* ≤ 0.05). *T*_o_, onset temperature; *T*_p_, peak temperature; *T*_c_, conclusion temperature; Δ*H*_gel_, gelatinization enthalpy; Δ*H*_ret_, retrogradation enthalpy.

**Table 4:** The Herschel–Bulkley rheological parameters of pineapple stem, cassava, corn, and rice starch gels.

Sample	Hysteresis Area (Pa/s)	*σ*_0_ (Pa)	*K* (Pa.s*^n^*)	*n* (−)	*R* ^2^
Pineapple stem	1850 ± 65.77 ^d^	6.51 ± 1.49 ^c^	2.017 ± 0.66 ^d^	0.614 ± 0.03 ^a^	0.987
Cassava	8941 ± 802.53 ^c^	1.70 ± 0.22 ^d^	7.608 ± 0.06 ^a^	0.295 ± 0.03 ^d^	0.978
Corn	34,950 ± 2126.62 ^b^	45.00 ± 1.78 ^b^	4.673 ± 0.40 ^b^	0.495 ± 0.01 ^c^	0.962
Rice	50,246 ± 2289.46 ^a^	56.40 ± 1.78 ^a^	3.153 ± 0.43 ^c^	0.536 ± 0.01 ^b^	0.960

Values in the same column with different superscripts are statistically different (*p* ≤ 0.05). *σ*_0_, yield stress; *K*, consistency coefficient; *n*, flow behavior index; *R*^2^, coefficient of determination.

**Table 5:** In vitro digestibility of cooked pineapple stem, cassava, corn, and rice starches.

Sample	RDS (%)	SDS (%)	RS (%)
Pineapple stem	35.38 ± 0.71 ^d^	48.84 ± 0.63 ^a^	15.77 ± 1.04 ^a^
Cassava	79.34 ± 0.16 ^a^	16.94 ± 0.86 ^d^	3.72 ± 0.70 ^c^
Corn	66.77 ± 0.24 ^b^	31.02 ± 0.27 ^b^	2.21 ± 0.39 ^d^
Rice	63.24 ± 0.24 ^c^	28.32 ± 0.39 ^c^	8.45 ± 0.63 ^b^

Values in the same column with different superscripts are statistically different (*p* ≤ 0.05). RDS, rapidly digestible starch; SDS, slowly digestible starch; RS, resistant starch.

## Data Availability

The data presented in this study are available on request from the corresponding author.
